# 3D hotspot matrix of Au nanoparticles on Au island film with a spacer layer of dithiol molecules for highly sensitive surface-enhanced Raman spectroscopy

**DOI:** 10.1038/s41598-021-01742-0

**Published:** 2021-11-17

**Authors:** Dong-Jin Lee, Dae Yu Kim

**Affiliations:** 1 Inha Research Institute for Aerospace Medicine, Inha University, Incheon, 22212 Republic of Korea; 2 Center for Sensor Systems, Inha University, Incheon, 22212 Republic of Korea; 3 Department of Electrical Engineering, College of Engineering, Inha University, Incheon, 22212 Republic of Korea

**Keywords:** Nanoscale materials, Optical materials and structures

## Abstract

Engineering of efficient plasmonic hotspots has been receiving great attention to enhance the sensitivity of surface-enhanced Raman scattering (SERS). In the present study, we propose a highly sensitive SERS platform based on Au nanoparticles (AuNPs) on Au island film (AuIF) with a spacer layer of 1,4-benzenedimethanethiol (BDMT). The three-dimensional (3D) hotspot matrix has been rationally designed based on the idea of employing 3D hotspots with a vertical nanogap between AuIF and AuNPs after generating large area two-dimensional hotspots of AuIF. AuNPs@BDMT@AuIF are fabricated by functionalizing BDMT on AuIF and then immobilizing AuNPs. The SERS performance is investigated with Rhodamine 6G as a probe molecule and the determined enhancement factor is 1.3 × 10^5^. The AuNPs@BDMT@AuIF are then employed to detect thiram, which is used as a fungicide, with a detection limit of 13 nM. Our proposed platform thus shows significant potential for use in highly sensitive SERS sensors.

## Introduction

SERS has been extensively studied and widely adopted in a diverse range of applications, including chemical identification, biomedical diagnosis, and environmental, food, and industrial monitoring^1–6^. SERS substrates enhance the naturally weak Raman scattering signals of target analytes by employing plasmonic hotspots in metallic nanostructures to boost and localize electromagnetic fields^1,7–9^. Traditionally, hotspot engineering has focused on the design of plasmonic building blocks, including materials and platforms with zero-dimensional to three-dimensional (3D) configurations^1,10–12^. Given the 3D laser excitation volume, 3D SERS platforms offer significant advantages over lower-dimensional SERS platforms. As such, various fabrication techniques have been proposed for the development of 3D SERS platforms, including top-down lithographic approaches and bottom-up self-assembly methods^6,8,13–20^.

Significant improvements in the efficient engineering of plasmonic hotspots have been achieved by means of nanofabrication technologies for realizing plasmonic nanogaps in high-performance SERS substrates^21^. In general, plasmonic nanogaps are classified into two types, horizontal nanogaps and vertical nanogaps. Horizontal nanogap architectures are made by a relatively simple fabrication process using traditional lithographic techniques such as ultraviolet lithography (UVL) and electron-beam lithography (EBL), therefore most studies have been conducted to develop horizontal nanogap structures typically with SERS enhancement factors (EFs) of the order of 10^5^–10^8^ ^21–23^. However, under typical lithography processes of UVL and EBL with spatial resolution limitations of sub-micron and sub-10 nm, respectively, it still remains a challenge to create horizontal plasmonic nanogaps at the sub-10 nm scale. In contrast to horizontal nanogaps, vertical nanogap architectures are possible to be manufactured in controlled sub-10 nm scale through bottom-up self-assembly processes.

Molecule-based self-assembly has fascinated attention towards highly sensitive SERS platforms due to their cost-effective fabrication and ease of precise control of nanogap^24–29^. A variety of molecules have been utilized to assemble nanoparticles (NPs) into 3D configurations. For example, Wu et al. demonstrated 3D plasmonic core-satellite nanostructures through a DNA molecule-assisted self-assembly^28^ and Solovyeva et al. used amino groups as a molecular linker for an assembly of silver NPs^26^. In addition, Lee et al. reported core-satellite gold NPs assembly with the use of dithiol molecules^29^. Linker molecules for the direct assembly of NPs enable the precise control of plasmonic hotspots, thus enhancing SERS performance.

In this study, we demonstrate a highly sensitive SERS platform based on gold NPs (AuNPs) on gold island film (AuIF) with a spacer layer of 1,4-benzenedimethanethiol (BDMT). Our proposed AuNPs@BDMT@AuIF SERS platform has been designed according to two distinct schemes: (1) in generating two-dimensional (2D) hotspots through large area AuIF, and (2) in employing 3D hotspot matrix by a vertical nanogap between AuIF and AuNPs. In our previous work, we have discussed the morphology change of 2D AuIF and the associated SERS enhancement according to the initial thickness of Au and the heat-treatment temperature^30^. Based on this study, the Au thin films with a thickness of ~ 4.5 nm were annealed at 550 °C for 10 min to fabricate the 2D AuIF. The BDMT was uniformly adsorbed onto the surface of the AuIF, leading to the self-assembly of nanometer-thick monolayers. The AuNPs@BDMT@AuIF SERS substrates were produced by immobilizing AuNPs with diameters of ~ 6–35 nm on the BDMT@AuIF. The SERS performance of the proposed platform was evaluated using rhodamine 6G (R6G). The Raman signal of the AuNP_35nm@BDMT@AuIF was up to twelve times stronger than that produced by AuIF at 100 nM of R6G. Furthermore, the proposed AuNPs@BDMT@AuIF platform was used to detect the fungicide thiram, exhibiting a limit of detection (LOD) of 13 nM.

## Results and discussion

**Figure 1:**
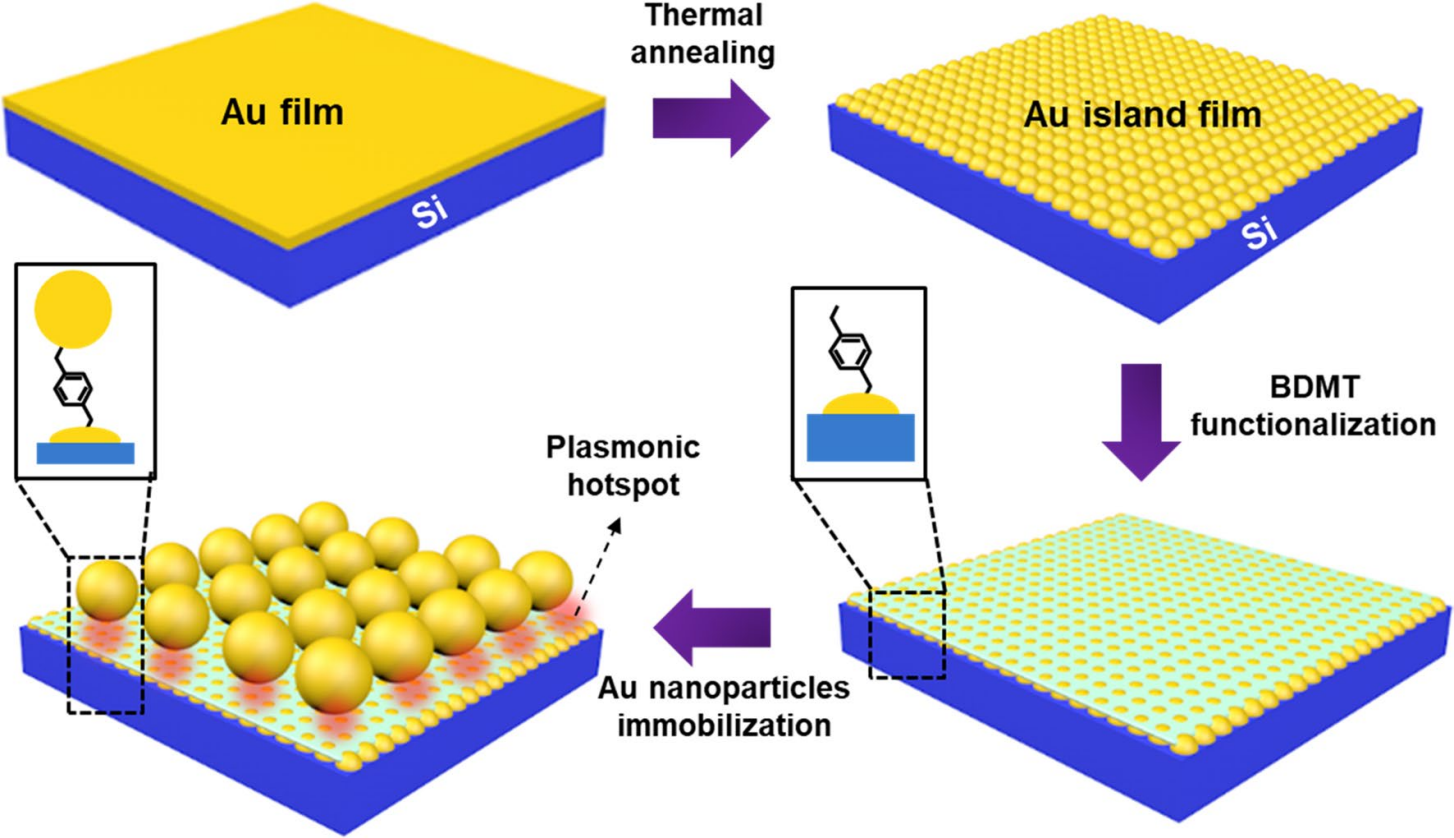
Schematic diagram of the fabrication process for AuNPs@BDMT@AuIF. AuIF is made by thermal annealing of Au thin film, and AuNPs@BDMT@AuIF are fabricated through immobilization of AuNPs on the BDMT@AuIF.

Figure 1 illustrates a step-by-step fabrication procedure for AuNPs@BDMT@AuIF sensors. Au thin film is deposited on the Si surface via sputtering then subjected to thermal annealing to produce 2D AuIF. After BDMT functionalization of the AuIF surface, AuNPs are immobilized on the BDMT@AuIF. BDMT has a chain length of approximately 1 nm and therefore provides a vertical nanogap between the AuIF and AuNPs^31^.

**Figure 2:**
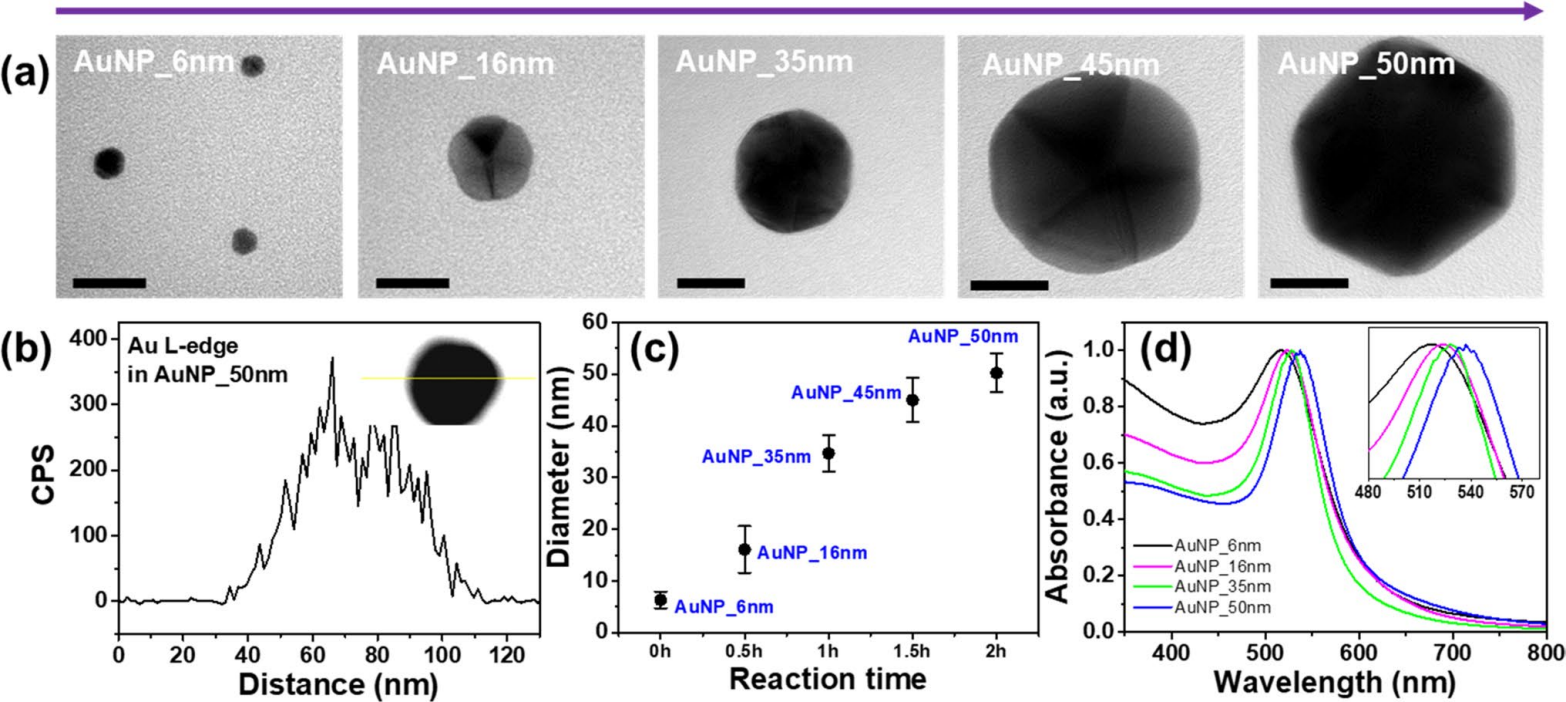
Morphological, chemical, and optical characteristics of the AuNPs. (**a**) TEM images of AuNPs according to reaction time. The scale bar is 20 nm. (**b**) EDS line-scan analysis of Au L-edge distribution across the AuNP_50nm. (**c**) Size distribution of the AuNPs as a function of reaction time. (**d**) UV–Vis absorption spectra of the AuNPs. The inset indicates a magnified view near the absorption peaks.

Figure 2 shows the morphological, chemical, and optical characteristics of AuNPs according to reaction time. As shown in Fig. 2a, the diameter of the AuNPs increased from 6.3 ± 1.6 nm (AuNP_6nm), to 16.1 ± 4.5 nm (AuNP_16nm), to 34.7 ± 3.6 nm (AuNP_35nm), to 44.9 ± 4.3 nm (AuNP_45nm), to 50.2 ± 3.7 nm (AuNP_50nm) according to reaction time. The lower-magnification TEM images of AuNPs were depicted in Fig. S2. To demonstrate the chemical elements of the AuNPs, EDS analyses were obtained for the AuNP_50nm. The line-scan analysis across the AuNP_50nm proved that Au L-edge distribution was well defined as shown in Fig. 2b. Figure 2c shows the diameter of AuNPs as an increase of reaction time. The detailed size distributions of AuNPs were depicted in Fig. S1. The TEM image analysis of the AuNPs was performed using freeware ImageJ (ver. 1.53e) program^32^. The optical properties of the AuNP solutions measured with UV–Vis spectroscopy are displayed in Fig. 2d. The absorption peak denotes the photon absorption due to the LSPR of the AuNPs, and the red-shift is observed as a successive particle growth, which is consistent with Mie theory^33^.

**Figure 3:**
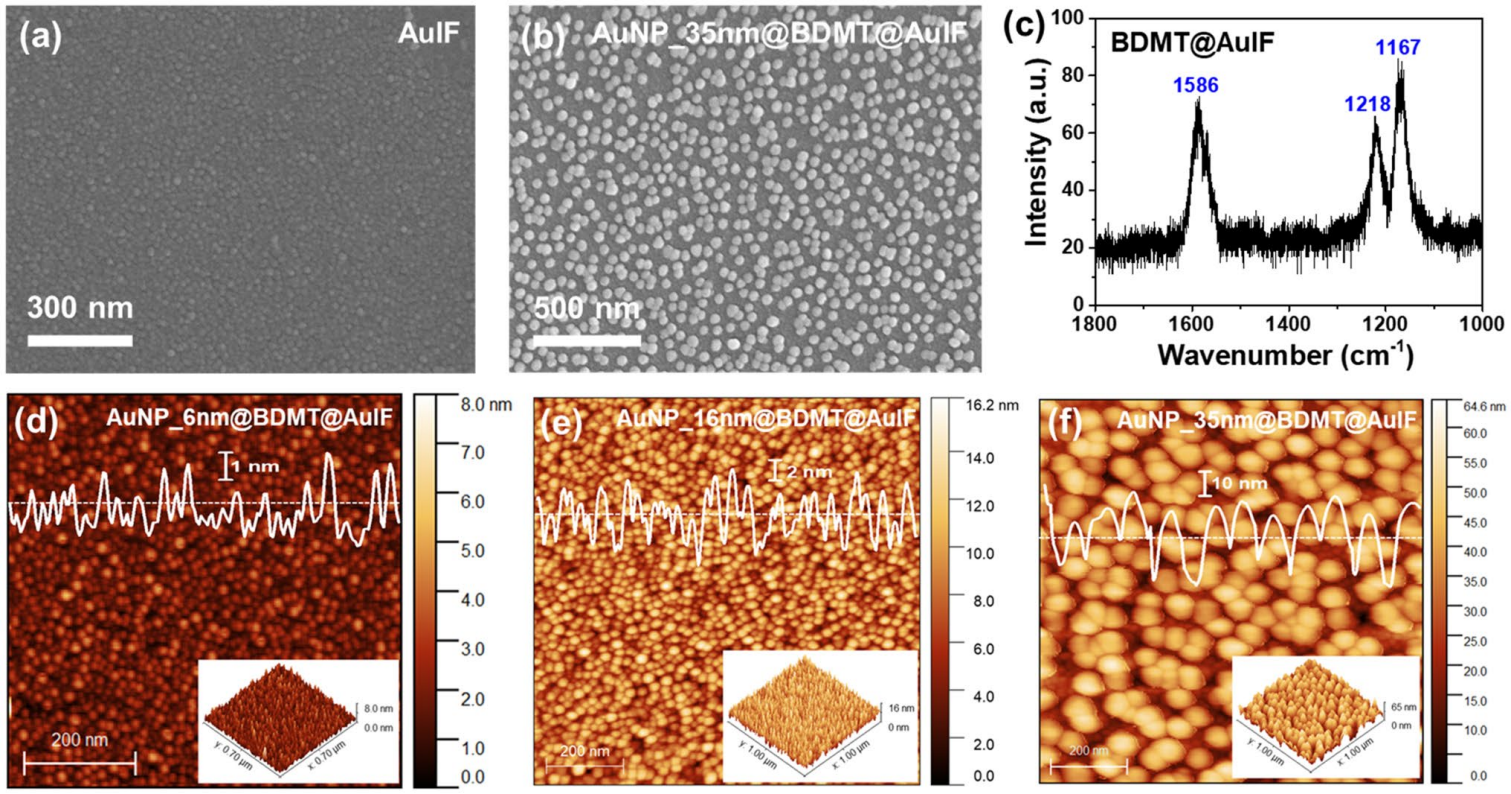
Morphological and chemical properties of the AuNPs@BDMT@AuIF. (**a**) and (**b**) SEM images of the AuIF and the AuNP_35nm@BDMT@AuIF, respectively. (**c**) Raman spectrum of the BDMT@AuIF. The characteristic peaks of 1586, 1218, and 1167 cm^**−**1^ corresponds to ring stretching, CH_2_ wagging, and β_C–H_ vibration modes of the BDMT SAMs, respectively. (**d**)–(**f**) AFM images of the AuNPs@BDMT@AuIF with different diameters of AuNPs. The white line profile inserted in each AFM image represents the height information along the line.

As shown in Fig. 2a, c, the difference in diameter between the AuNP_45nm (44.9 ± 4.3 nm) and the AuNP_50nm (50.2 ± 3.7 nm) is about 10%. To establish clear and concise experimental conditions according to the size of AuNPs, we performed subsequent experiments except for the AuNP_45nm. Figure 3 shows the morphological and chemical properties of the AuNPs@BDMT@AuIF. Figure 3a and b present SEM images of the AuIF and the AuNP_35nm@BDMT@AuIF, respectively. After the thermal annealing process of the Au thin film, 2D AuIF with a hemispherical surface was obtained as the grain size of the thin film increases. Following the immobilization of the AuNPs, AuNPs@BDMT@AuIF substrates were obtained as shown in Fig. 3b and d–f. However, the AuNP_50nm was not well immobilized on the BDMT@AuIF surface presumably due to a fast sedimentation, strong friction against the substrate, and difficulty in maintaining the uniformity of the AuNPs during self-assembly process (Fig. S3)^34^. To verify the functionalization of the well-ordered BDMT SAMs, the surface-enhanced Raman spectrum of the BDMT@AuIF was measured. The characteristic peak at 1586 cm^−1^ is identified as the ring stretching mode, and the peaks at 1218 and 1167 cm^−1^ indicate the CH_2_ wagging mode and the β_C-H_ vibration mode, respectively^35,36^. Figure 3d–f shows AFM images of the AuNP_6nm@BDMT@AuIF, the AuNP_16nm@BDMT@AuIF, and the AuNP_35nm@BDMT@AuIF, respectively. The 3D topographic image and height profile along the line were inserted in each AFM image. From the AFM images, it can be seen that AuNPs with diameters of ~ 6–35 nm were moderately immobilized on the surface of BDMT@AuIF.

**Figure 4:**
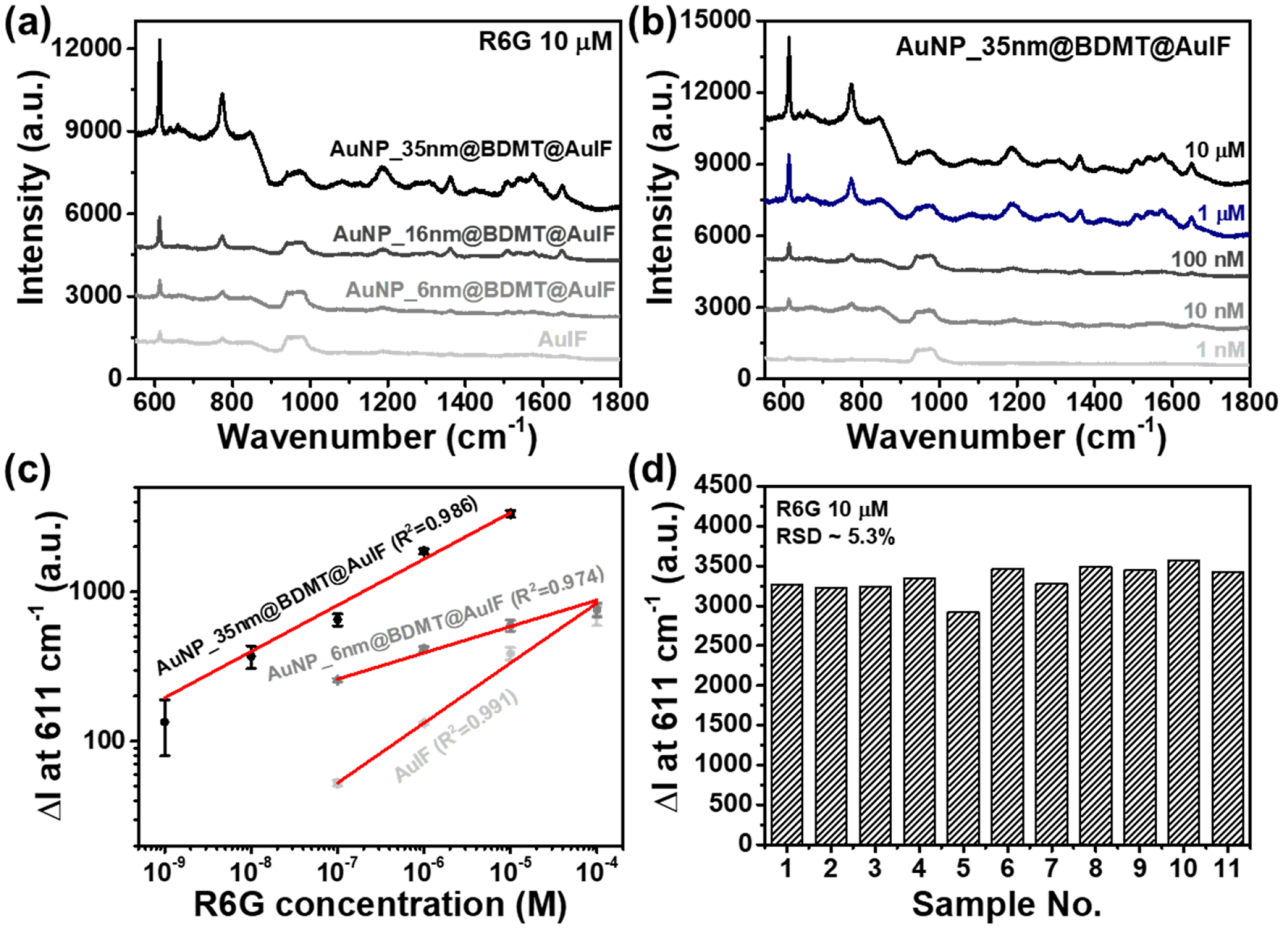
SERS performance of the AuNPs@BDMT@AuIF for R6G molecules. (**a**) Raman spectra for the AuIF and the AuNPs@BDMT@AuIF with different diameters of AuNPs at 10 μM of R6G concentration. The Raman signal of the AuNP_35nm@BDMT@AuIF sensor is about eight times higher than that of AuIF sensor. (**b**) SERS spectra of the AuNP_35nm@BDMT@AuIF sensor at R6G concentrations of 10^**−**5^ to 10^**−**9^ M. (**c**) Raman intensity at 611 cm^**−**1^ as a function of the logarithmic concentration of R6G for the AuIF, the AuNP_6nm@BDMT@AuIF, and the AuNP_35nm@BDMT@AuIF sensors. (**d**) Intensity distribution at 611 cm^**−**1^ was evaluated for 11 different regions at 10 μM of R6G concentration for the AuNP_35nm@BDMT@AuIF sensor.

To evaluate the SERS performance of the proposed AuNPs@BDMT@AuIF sensors, the SERS signals of the AuNPs@BDMT@AuIF sensors were measured using R6G molecules at a range of concentrations (10^−4^ to 10^−9^ M). The R6G spectra exhibited strong peaks for vibrational bands at 611, 773, 1181, 1310, 1360, 1507, 1574, and 1648 cm^−1^, corresponding to the Raman characteristic peaks of R6G, as shown in Fig. S4 and Table S2. Figure 4a presents the Raman spectra for the AuIF and the AuNPs@BDMT@AuIF with different diameters of AuNPs at 10 μM R6G. It is obvious that the Raman intensities of the AuNP_35nm@BDMT@AuIF sensor are strongly enhanced, which is about eight times higher than that of the AuIF sensor. Figure 4b shows the SERS spectra of the AuNP_35nm@BDMT@AuIF sensor at R6G concentrations of 10^−5^ to 10^−9^ M. Figure S5a–c shows the SERS spectra of the AuIF, the AuNP_6nm@BDMT@AuIF, and the AuNP_16nm@BDMT@AuIF sensors at R6G concentrations of 10^−4^ to 10^−7^ M, respectively. Figure 4c presents the Raman intensity at 611 cm^−1^ as a function of the logarithmic concentration of R6G for the AuIF, the AuNP_6nm@BDMT@AuIF, and the AuNP_35nm@BDMT@AuIF sensors (SERS data of the AuNP_16nm@BDMT@AuIF sensor is separately presented in Fig. S5d for clarity). For the AuNP_35nm@BDMT@AuIF sensor, the signal increase ($\Delta I$) of the Raman peak at 611 cm^−1^ shows a good linear relationship with R6G logarithmic concentration ($C_{R6G}$), which can be expressed by $Log(\Delta I) = 0.30991Log(C_{R6G} ) + 5.07964$ having an R^2^ of 0.986. The SERS signal of the AuNP_35nm@BDMT@AuIF sensor at 611 cm^−1^ increased by about 12 times compared to the AuIF sensor at 100 nM of R6G concentration. The limit of detection (LOD) was computed according to the formula of $LOD = mean_{blank} + 1.645(SD_{blank} + SD_{analyte} )$, where $mean_{blank}$ and $SD_{blank}$ are the average and the standard deviation of $\Delta I$ without analyte, respectively, and $SD_{analyte}$ is the standard deviation of $\Delta I$ for the lowest analyte concentration measured^37^. The obtained LOD was about 10^−10.07^ M (~ 84 pM). To investigate the reproducibility of the AuNP_35nm@BDMT@AuIF sensor, the Raman intensities at 611 cm^−1^ were evaluated for 11 different regions at 10 μM of R6G concentration as shown in Fig. 4d, and the relative standard deviation (RSD) was about 5.3%. Figure S6a shows the Raman intensity at 773 cm^−1^ as a function of the logarithmic concentration of R6G for the AuNP_35nm@BDMT@AuIF sensors with an R^2^ of 0.931, and the intensity distribution was depicted in Fig. S6b. The RSD was about 5.2%.

To quantitatively compare the SERS performance, the SERS enhancement factor (EF) was calculated from the Raman intensity of peak at 611 cm^−1^ of R6G molecules using the following equation ^38–41^:

$$ EF = \frac{{I_{SERS} }}{{I_{bulk} }} \times \frac{{N_{bulk} }}{{N_{SERS} }} $$

where $I_{SERS}$ and $I_{bulk}$ are the Raman intensities at 611 cm^−1^ for the AuNP_35nm@BDMT@AuIF and bulk sample, respectively; $N_{bulk}$ and $N_{SERS}$ are the number of R6G molecules adsorbed on the Si surfaces and the AuNP_35nm@BDMT@AuIF surfaces, respectively. To decide $N_{bulk}$, 10 μL of R6G solution at a concentration of 1 mM was spread onto a Si substrate. $N_{SERS}$ was determined by the R6G drop solution (1 nM, 10 μL) on the AuNP_35nm@BDMT@AuIF sample. The ratio of was calculated to be 10^6^ under the same area of laser spot. Raman spectra for the two samples were observed under the same conditions. The ratio of $I_{SERS} /I_{bulk}$ was calculated to be 0.13 from the Raman intensities at 611 cm^−1^, and we determined the EF value of 1.3 × 10^5^ for our proposed AuNP_35nm@BDMT@AuIF sample. As shown in Table S3, this value is lower than the EF obtained by Au nano-islands^38^ and is comparable to some EFs enabled by DNA-Au nanowire structures^39^ and silver dendrites^40^.

**Figure 5:**
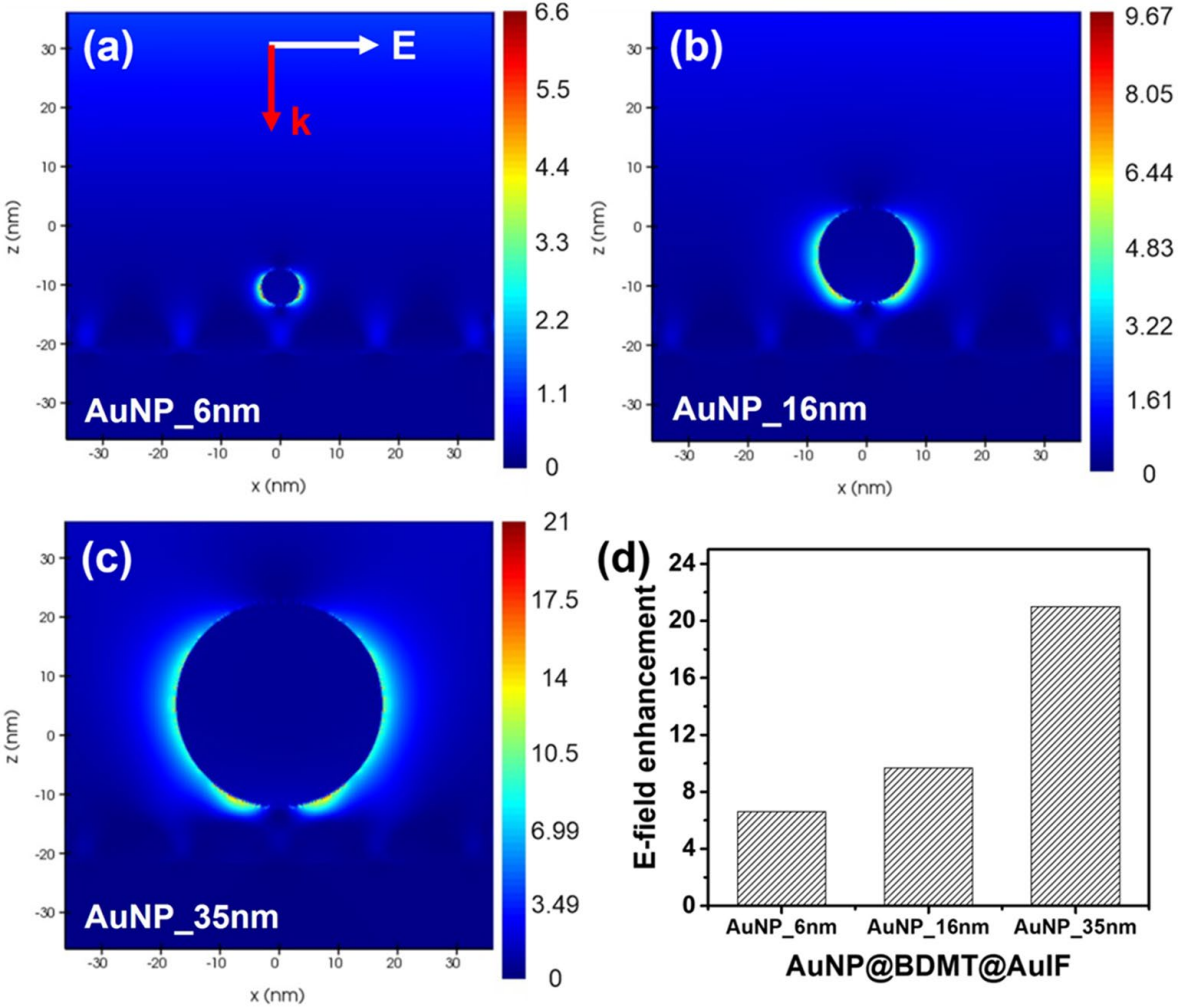
FDTD simulations illustrating E-field localization and enhancement at AuNP_6nm (**a**), AuNP_16nm (**b**), and AuNP_35nm (**c**). (**d**) E-field enhancement for different size of the AuNPs. (Inset of **a**) White arrow indicates the E-field direction and red arrow depicts the direction of propagation.

To evaluate the electric field distribution of the proposed AuNP@BDMT@AuIF sensor, the finite-difference time-domain (FDTD) simulation was used to calculation the electric field enhancement of the proposed AuNP@BDMT@AuIF (3D FDTD-Lumerical, Ansys, Waterloo, ON N2J 4G8, Canada)^42^. In the FDTD modelling, the AuIF was approximated to a hemisphere array with a period of 16 nm (Fig. S8). The diameter and step height of each hemisphere were set to 20.7 nm and 4.3 nm, respectively as shown in the Fig. S7. Each AuNP with different diameters of 6.3 nm (AuNP_6nm), 16.1 nm (AuNP_16nm), and 34.7 nm (AuNP_35nm) was placed on the AuIF/Si substrate along the z-direction. In addition, the closest distance between AuNP and AuIF was set to 1 nm to represent a vertical nanogap by the BDMT monolayer. During the simulations, a total-field scattered-field (TFSF) source polarized in the x-direction is injected into a box in the wavelength range from 400 to 700 nm, and perfectly matched layer (PML) absorbing boundary conditions were utilized. Figure 5 shows the simulated electric field (E-field) distributions of the AuNP_6nm, the AuNP_16nm, and the AuNP_35nm on the AuIF/Si substrate following irradiation with 532 nm light polarized in the x-direction, respectively. The E-field enhancement was calculated from (E/E_inc_)^2^, where E_inc_ is the E-field of the incident light, and E is the E-field near the AuNP. Figure S9 shows the FDTD simulation results of the E-field distribution of the 2D hotspots by the AuIF. The E-field enhancement of the AuIF showed a rather weak value of 1.518. However, the E-field enhancement of the 3D hotspots by the AuNP on AuIF/Si substrate increased significantly to 6.6, 9.67, and 21 as the diameter of the AuNP increased to 6.3, 16.1, and 34.7 nm, respectively. As shown in the Fig. 5a–c, it can be seen that more E-fields were concentrated in the plasmonic nanogaps between the AuNP and the AuIF. In addition, the highest electric field enhancement was achieved at a AuNP_35nm, which is ~ 3.2 times as strong as that of AuNP_6nm.

**Figure 6:**
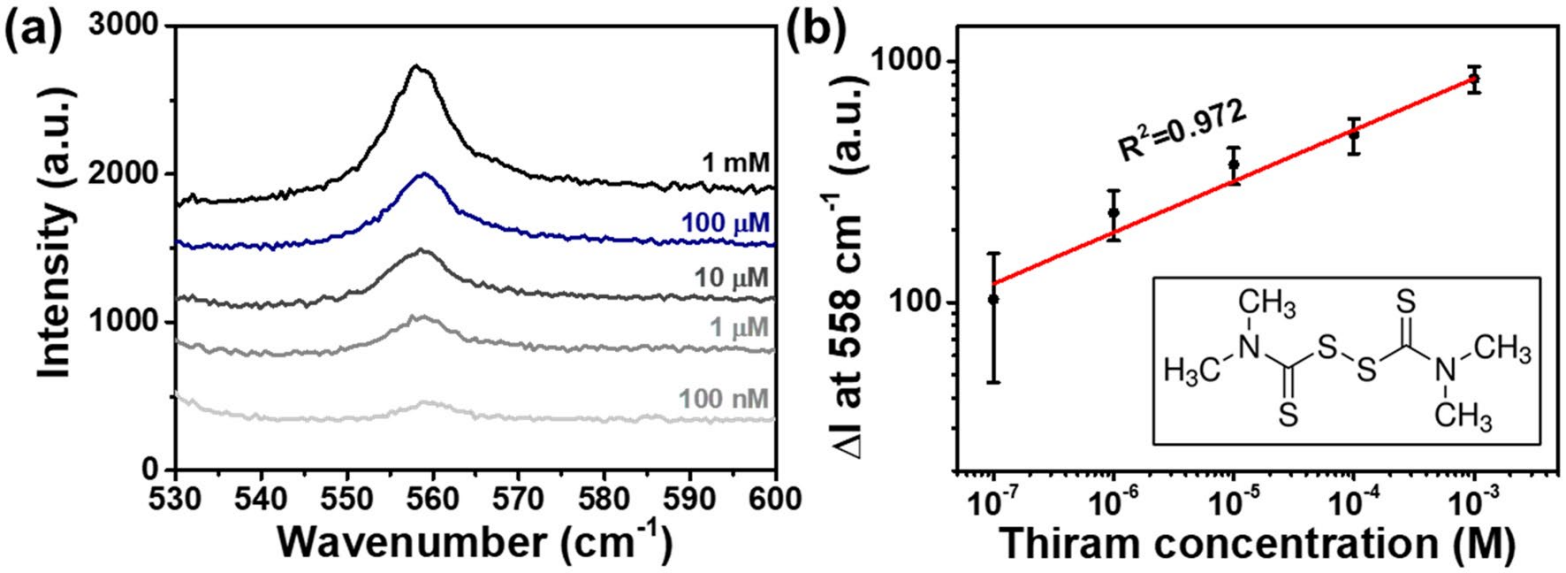
SERS enhancement of the AuNP_35nm@BDMT@AuIF for thiram. (**a**) SERS spectra of the AuNP_35nm@BDMT@AuIF sensor at thiram concentrations of 10^**−**5^ to 10^**−**7^ M. (**b**) Raman intensity at 558 cm^**−**1^ as a function of the logarithmic concentration of thiram. The inset indicates the molecular structure of thiram.

To demonstrate the practical utility of our proposed sensing platform, we investigated the SERS performance of the AuNP_35nm@BDMT@AuIF with thiram. As a kind of sulfur fungicide, thiram has been widely utilized in agricultural production as a foliar treatment on fruits, vegetables, and food crops^43–45^. Thiram has been also used in human scabies treatment as a sunscreen and a bactericide^45^. Due to its toxicity, excessive abuse of thiram leads to the environmental pollution and raises many issues concerning its impact on human health such as eyes, skin, and respiratory tract^43,44^. Figure 6a shows the SERS spectra of the AuNP_35nm@BDMT@AuIF sensor at thiram concentrations of 10^−3^ to 10^−7^ M. Figure 6b presents the signal increase ($\Delta I$) at 558 cm^−1^ as a function of the logarithmic concentration of thiram ($C_{Thiram}$) described by $Log(\Delta I) = 0.1957Log(C_{Thiram} ) + 3.5109$ with the R^2^ of 0.972. The LOD was found to be ~ 13 nM. Compared to the recently reported SERS substrates, this LOD value is compatible to that of other SERS substrates as depicted in Table S4. In addition, the LOD value is about 1000 times lower than the maximal residue limit of 7 ppm (~ 29 μM) in fruit^46^. These results indicate that our proposed SERS platform shows satisfactory performance and suggests an alternative approach for highly sensitive SERS sensor.

## Conclusions

We demonstrated the rationally designed AuNPs@BDMT@AuIF SERS platform based on the idea of employing 3D hotspots with a vertical nanogap between the AuIF and the AuNPs after generating 2D hotspots in large area AuIF. We evaluated the fabricated sensor using SEM, TEM, AFM, EDS, and UV–vis spectrometer. The SERS signals of R6G concentrations 100 μM to 1 nM were obtained from the AuNPs@BDMT@AuIF with different size of AuNPs, and it was found that the SERS EF was 1.3 × 10^5^. We revealed that these results were due to the strong electric fields in the nanogap between the AuNPs and the AuIF via 3D FDTD simulation. In addition, our proposed sensor was able to measure thiram up to 13 nM, showing significant potential for use in highly sensitive SERS sensors.

## Methods

### Materials

Silicon wafers were purchased from Silicon Materials Inc., USA (B-doped p-type wafer, resistivity of 1–30 Ω∙cm). Rhodamine 6G, gold chloride trihydrate, 1,4-benzenedimethanethiol, n-hexane, sodium citrate, and thiram were purchased from Sigma-Aldrich. The Au target was purchased from Thifine Inc. (2-inch diameter, purity of 99.99%). All chemicals required no further purification.

### Synthesis of AuNPs with a diameter of 6–50 nm

AuNPs in aqueous suspension were obtained by seed-mediated synthesis according to the previous report^47^. Briefly, to synthesize Au seeds, 1 mL of 25 mM gold chloride trihydrate aqueous solution was injected to 150 mL of 2.2 mM sodium citrate aqueous solution at 100 ℃ under vigorous stirring. After 10 min, the color of the Au seeds changed from yellow to red-wine. For the synthesis of AuNPs up to 50 nm in diameter, the reaction suspension was allowed to cool down to 90 °C. Then, 1 mL of 60 mM sodium citrate aqueous solution and 1 mL of 25 mM gold chloride trihydrate aqueous solution were added to the same vessel in consecutive order and kept for 30 min. By repeating this process four times, AuNPs with a diameter of 6–50 nm were obtained. For convenience and clarity, we denoted Au seed as AuNP_6nm, AuNPs with different diameters as AuNP_16nm to AuNP_50nm. Table S1 indicates the information of size and localized surface plasmon resonance (LSPR) peak wavelength in AuNPs according to reaction time. Milli-Q water was used for the preparation of all aqueous solutions.

### Preparation of AuNPs@BDMT@AuIF

The 2D AuIF were prepared following the procedure described in our previous study^30^. Briefly, Au thin films with a thickness of 4.5 nm were deposited on the Si wafers, and then thermally annealed at temperatures of 550 ℃ for 10 min. AuNPs@BDMT@AuIF sensors for SERS measurement were obtained as follows. In order to get well-ordered self-assembled monolayers (SAMs) of BDMT, AuIF were immersed in a freshly prepared 1 mM BDMT solution of *n*-hexane for 30 min at 60 °C and then rinsed with a fresh solution of n-hexane^48^. The as-prepared BDMT@AuIF were subsequently washed with large volumes of water and ethanol and then immersed in an AuNPs suspension having different diameters overnight at 4 °C^49^. The resulting AuNPs@BDMT@AuIF were rinsed again with water before analysis.

### Characterization techniques

The morphological and chemical information of the fabricated samples were characterized using scanning electron microscopy (SEM; Hitachi S-4300SE, Hitachi, Japan), transmission electron microscopy (TEM; JEM-2100F, JEOL, Japan), atomic force microscopy (AFM; NanoScope IV, Bruker, MA, USA), and Fourier transform-infrared spectrometer (FT-IR; Vertex 80 V, Bruker, MA, USA). Elemental mapping was carried out using Thermo-Noran energy dispersive X-ray spectroscopy (EDS) attachment equipped with TEM (JEM-2100F). UV–Vis spectrometer (Lambda 650, PerkinElmer, MA, USA) was utilized for optical characterization of the synthesized AuNPs.

### SERS measurements

For monitoring of SERS behaviors to R6G, AuIF samples and AuNPs@BDMT@AuIF samples were immersed in an aqueous solution of R6G with different concentrations ranging from 100 μM to 1 nM for 1 h, as described in our previous studies^30,50^. The samples were then rinsed in Milli-Q water and dried with a nitrogen blow to remove the unfixed molecules. For SERS measurements of thiram, an ethanolic solution of thiram (1 mM–100 nM) was dropped on AuNPs@BDMT@AuIF samples and then dried naturally. Raman spectra for R6G were collected on a Raman spectroscopy (LabRAM HR Evolution, HORIBA, Japan) under the conditions of an excitation wavelength of 532 nm, a laser power of 14 mW, an acquisition time of 5 s, and an accumulation of 3 in a range of 400–1800 cm^−1^. SERS spectra for thiram were acquired at an excitation wavelength of 532 nm, a laser power of 14 mW, an acquisition time of 10 s, and an accumulation of 5 in a range of 400–1800 cm^−1^.

## Supplementary Information

Supplementary Information.
