# Supplementary material for: 3D hotspot matrix of Au nanoparticles on Au island film with a spacer layer of dithiol molecules for highly sensitive surface-enhanced Raman spectroscopy
Source: Sci Rep. 2021 Nov 17;11:22399. doi: 10.1038/s41598-021-01742-0 (PMC8599516; doi:10.1038/s41598-021-01742-0)
Supplement: Supplementary file 1 — Supplementary Information. [file 41598_2021_1742_MOESM1_ESM.pdf]

Supporting Information for

3D Hotspot Matrix of Au Nanoparticles on Au Island Film with a Spacer Layer of Dithiol Molecules for Highly Sensitive Surface-Enhanced Raman Spectroscopy

Dong-Jin Lee[1,2] *and Dae Yu Kim*[1,2,3]*

[1]*Inha Research Institute for Aerospace Medicine,* [2]*Center for Sensor Systems,* [3]*Department of Electrical Engineering, College of Engineering, Inha University, Incheon 22212, Republic of Korea*

KEYWORDS: SERS; AuNPs@BDMT@AuIF; 3D Hotspot Matrix; Vertical configuration with nanogap; Spacer layer of dithiol molecules; Pesticide detection

**Corresponding Author: dyukim@inha.ac.kr*

1. Size and optical properties of AuNPs according to reaction time

Table S1. Size and localized surface plasmon resonance (LSPR) peak wavelength of AuNPs according to reaction time.

AuNPs	Diameter (nm)	Standard deviation (nm)	LSPR peak (nm)
AuNP_6nm	6.32589	1.61402	517
AuNP_16nm	16.0919	4.50535	523
AuNP_35nm	34.67985	3.58913	528
AuNP_45nm	44.9785	4.29666	-
AuNP_50nm	50.18752	3.70241	537

2. Size distribution of AuNPs

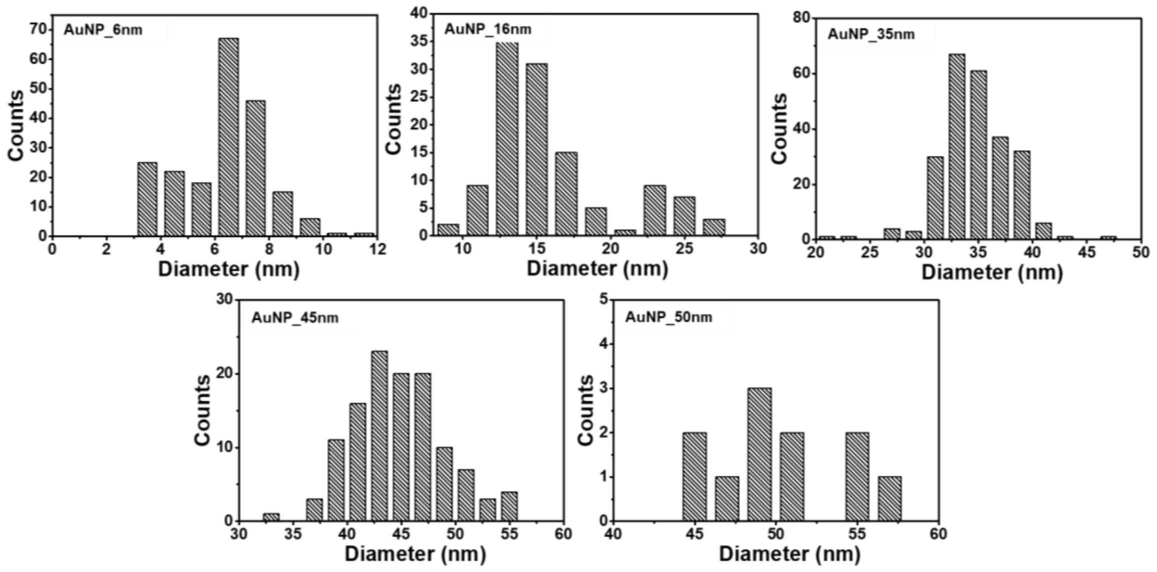

Figure S1. Size distribution of the AuNPs according to reaction time.

3. TEM images and EDS spectrum of AuNPs

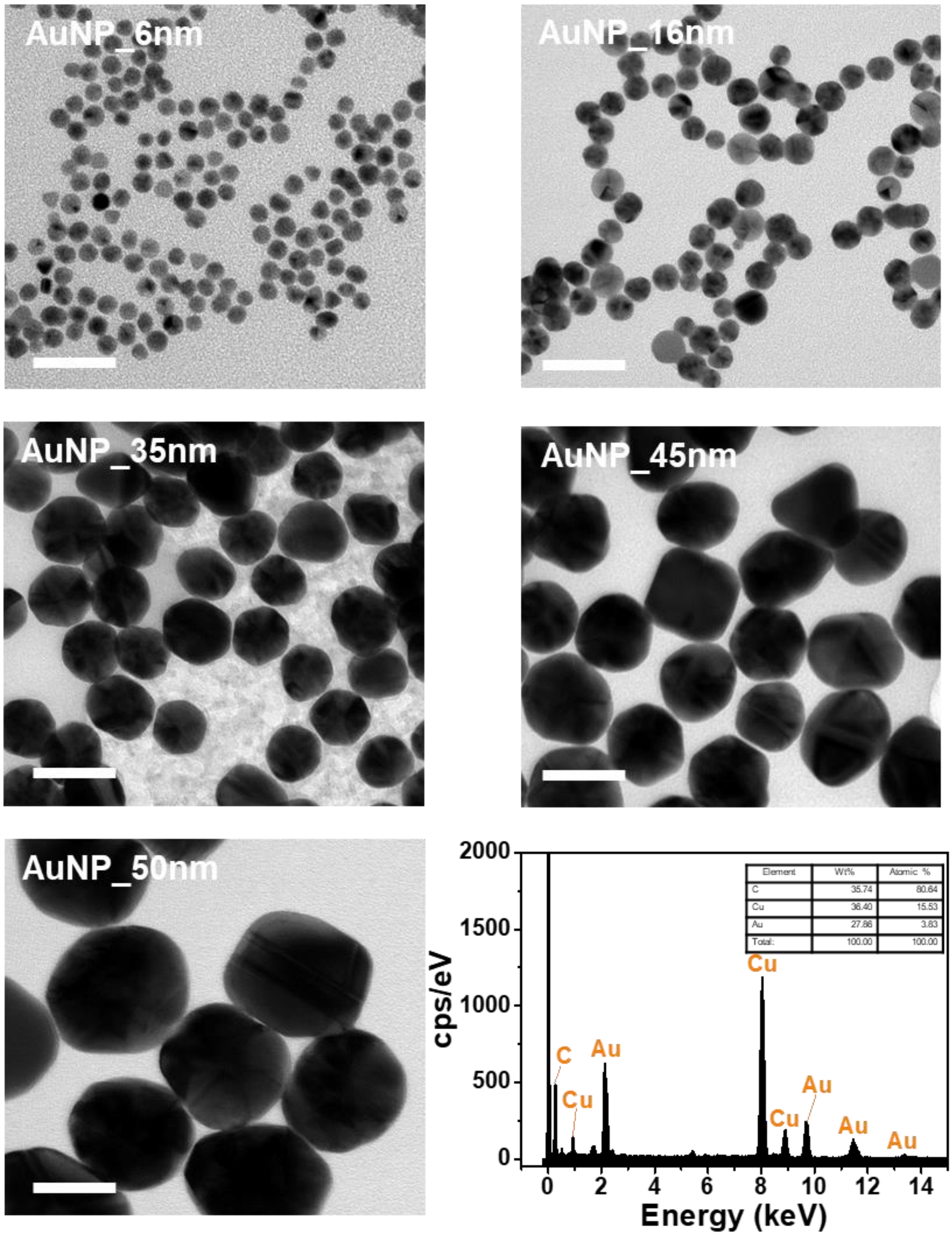

Figure S2. TEM images and EDS spectrum of the AuNPs.

4. SEM image of the AuNP_50nm@BDMT@AuIF

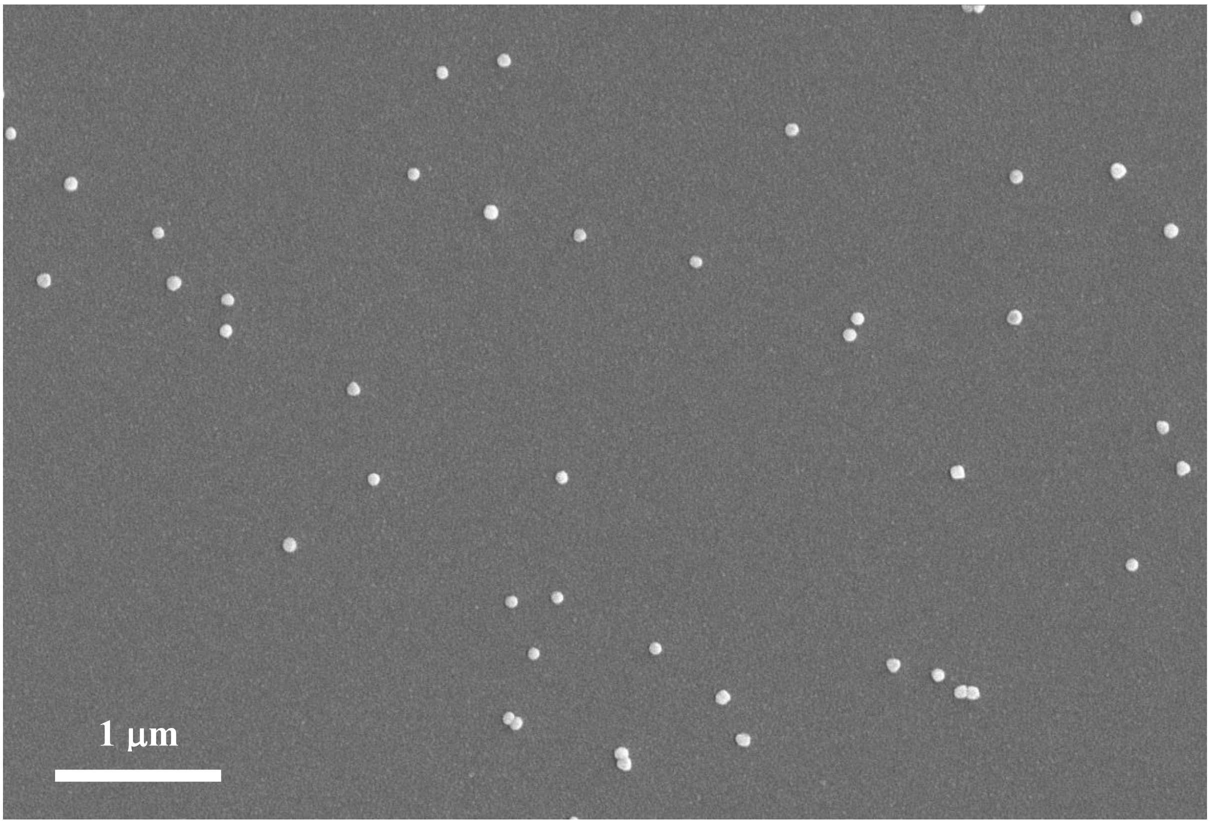

Figure S3. SEM image of the AuNP_50nm@BDMT@AuIF. The AuNP_50nm was not well immobilized on the BDMT@AuIF surface.

5. Raman characteristic peaks of R6G

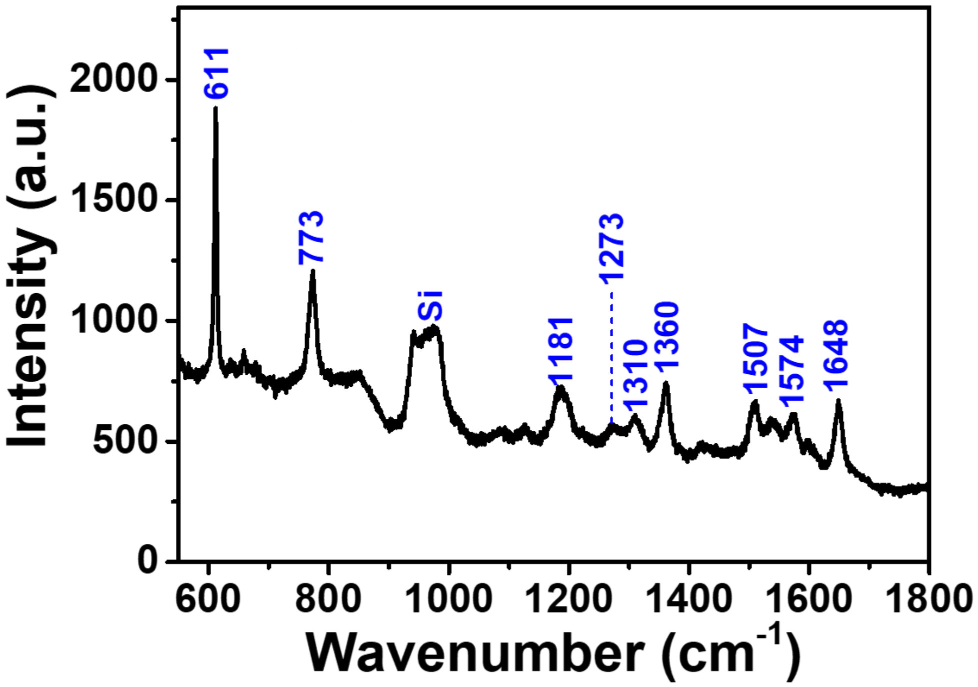

Figure S4. Raman spectrum of R6G.

Table S2. Peak positions and assignments of the Raman spectrum of R6G.

Wavenumber (cm^{-1})	Assignment
**611**	**In plane xanthene ring deformations; Out of plane xanthene ring deformations**
**773**	**Out of plane C-H bend; In plane xanthene ring deformations**
**1181**	**In plane xanthene ring deformations; In plane C-H bend; In plane N-H bend**
**1310**	**In plane xanthene ring breath; In plane N-H bend; CH_2 wag**
**1360**	**Xanthene ring stretch; In plane C-H bend**
**1507**	**Xanthene ring stretch; C-N stretch; C-H bend; N-H bend**
**1574**	**Xanthene ring stretch; in plane N-H bend**
**1648**	**Xanthene ring stretch; in plane C-H bend**

6. SERS performance of the proposed AuNP@BDMT@AuNSs

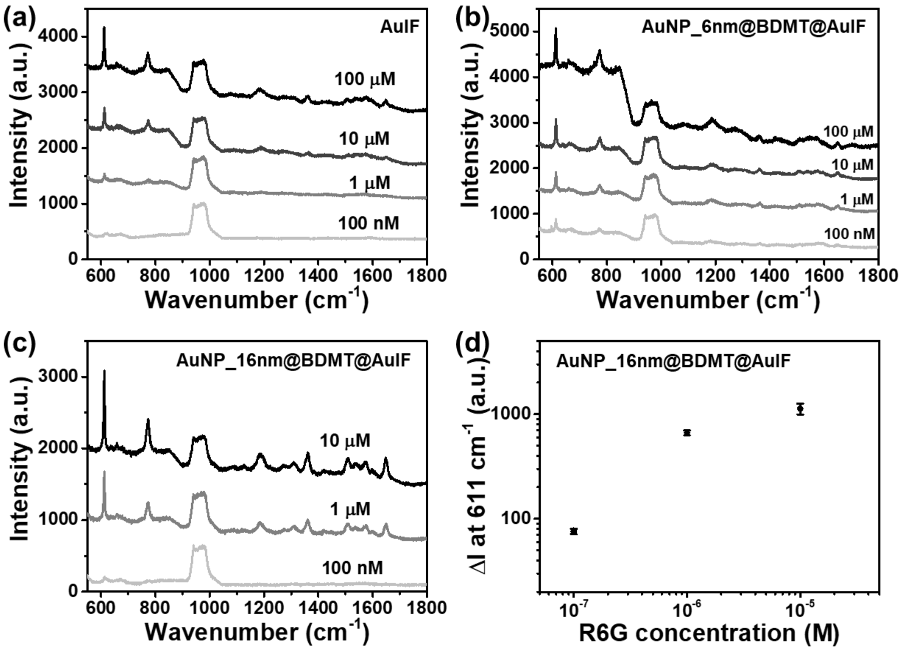

Figure S5. SERS performance of AuIF and AuNP@BDMT@AuIF for R6G. SERS spectra of (a) the AuIF, (b) AuNP_6nm@BDMT@AuIF, and (c) AuNP_16nm@BDMT@AuIF sensor at R6G concentrations of 10^{-5} to 10^{-7} M. (d) Raman intensity at 611 cm^{-1} as a function of the logarithmic concentration of R6G for the AuNP_16nm@BDMT@AuIF sensor.

7. SERS performance of the proposed AuNP_35nm@BDMT@AuIF

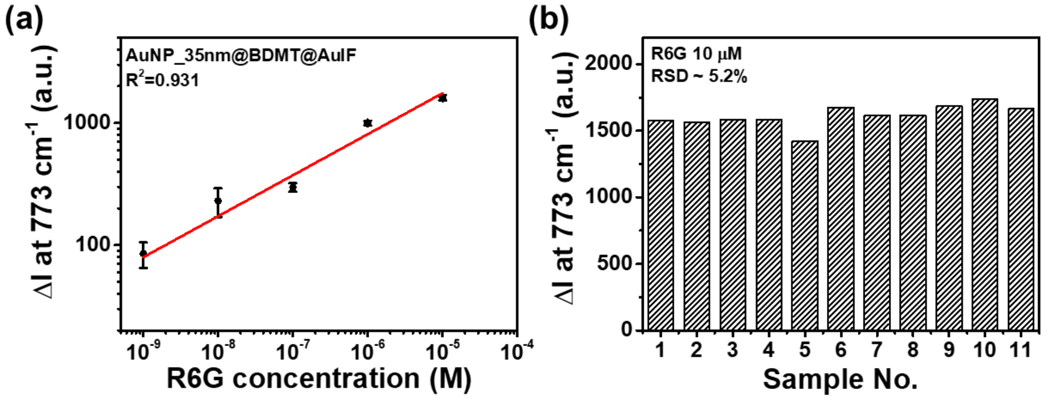

Figure S6. SERS performance of the AuNP_35nm@BDMT@AuIF for R6G. (a) Raman intensity at 773 cm^{-1} as a function of the logarithmic concentration of R6G for the AuNP_35nm@BDMT@AuIF sensor. (b) Raman intensity at 773 cm^{-1} was evaluated for 11 different regions at 10 μM of R6G concentration for the AuNP_35nm@BDMT@AuIF sensor.

8. FDTD simulation model

Figure S7 shows the structural details of Au island film obtained by AFM measurements and freeware ImageJ (ver. 1.53e) analysis. It was found that the Au islands had a hemispherical shape, and the mean diameter and step height were 20.7 nm and 4.3 nm, respectively.

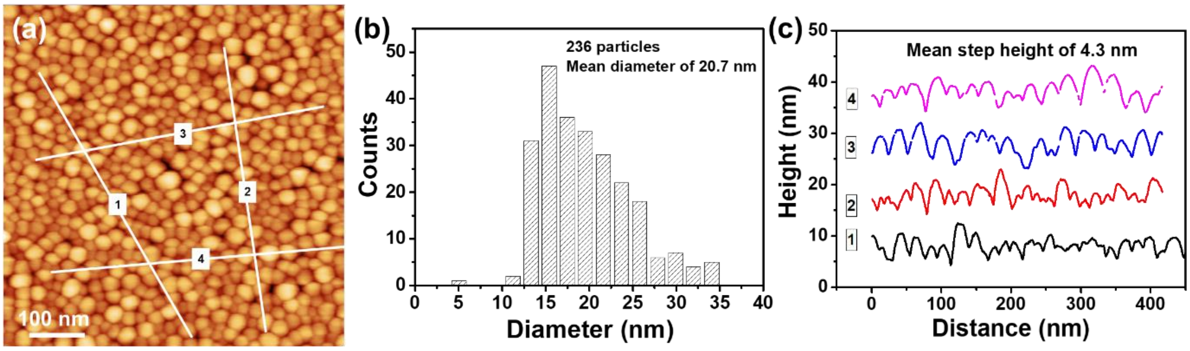

Figure S7. Structural details of Au island film. (a) AFM image of Au island film. (b) Particle distribution obtained by ImageJ analysis. (c) Line profiles from different regions in Fig. S7(a).

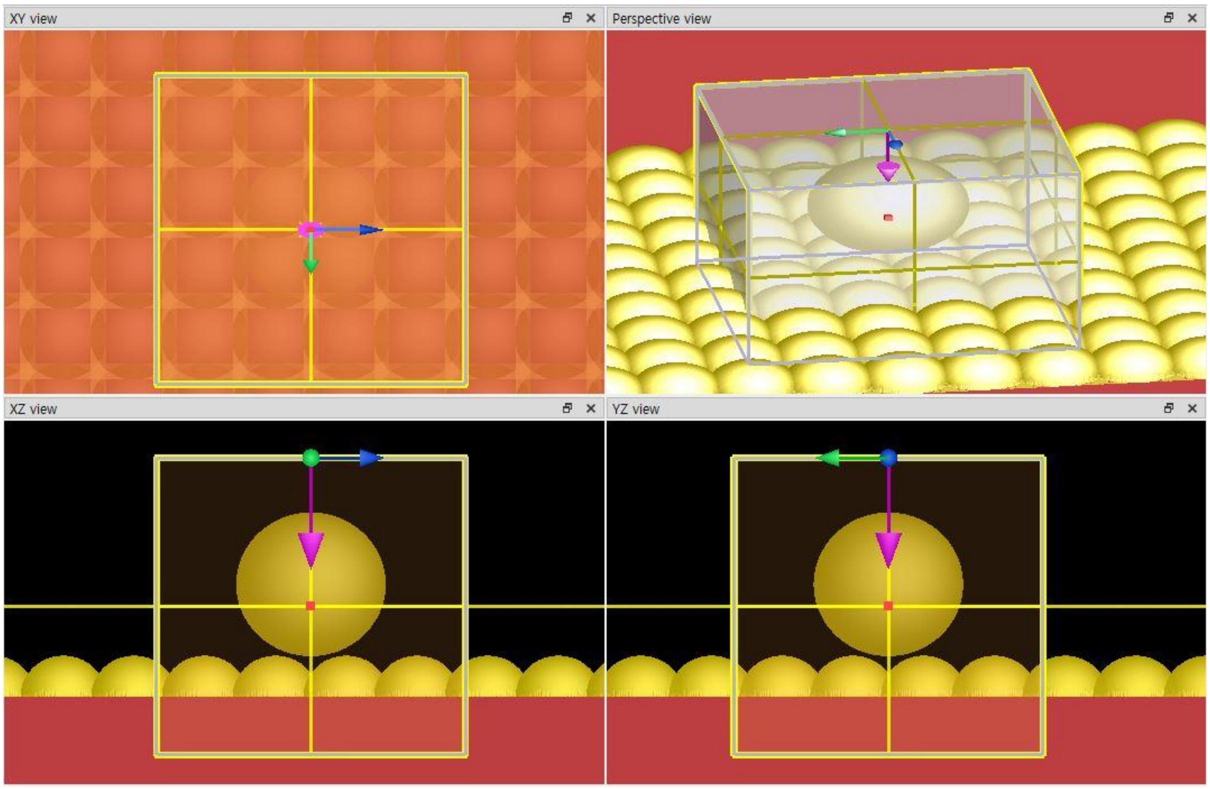

Figure S8. Structural model of the AuNP_35nm@BDMT@AuIF in the FDTD simulation.

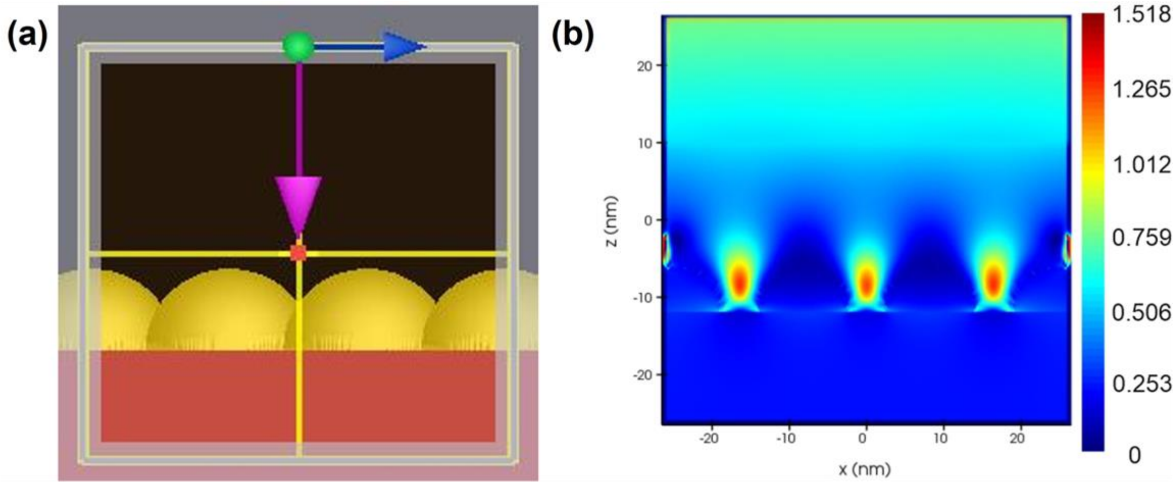

Figure S9. Structural model (a) and E-field distribution (b) of the AuIF in the FDTD simulation.

9. EF comparison of recent SERS substrates for R6G

Table S3. EF comparison of recent SERS substrates for R6G.

SERS substrate	EF	References
Au nano-islands	10^7-10^8	[1]
DNA–Au nanowire structures	10^5-10^6	[2]
Silver dendrites	4×10^5	[3]
Au colloids assembly	1.5×10^3	[4]
AuNP@BDMT@AuNSs	1.3×10^5	This work

10. LOD comparison of recent SERS substrates for thiram

Table S4. LOD comparison of recent SERS substrates for thiram.

SERS substrate	LOD	References
Silver nanodendrites	1.2 ppb (5 nM)	[5]
Flexible PET/ITO/Ag	0.16 μg/mL (0.67 nM)	[6]

SiO2@Au@4-MBA@Ag nanomaterials	72 ppb (300 nM)	[7]
2D Au@Ag Nanorods array	75 nM	[8]
Bimetallic core shelled nanoparticles (Au@AgNPs)	60 ppb (250 nM)	[9]
AuNP@BDMT@AuIF	13 nM	This work

[References]

[1] Fusco, Z. et al. Self-assembly of Au nano-islands with tuneable organized disorder for highly sensitive SERS. Journal of Materials Chemistry C 7, 6308-6316, doi:10.1039/c9tc01231a (2019).

[2] Kundu, S. & Jayachandran, M. The self-assembling of DNA-templated Au nanoparticles into nanowires and their enhanced SERS and catalytic applications. RSC Adv. 3, doi:10.1039/c3ra42203h (2013).

[3] Dies, H., Raveendran, J., Escobedo, C. & Docoslis, A. In situ assembly of active surface-enhanced Raman scattering substrates via electric field-guided growth of dendritic nanoparticle structures. Nanoscale 9, 7847-7857, doi:10.1039/c7nr01743j (2017).

[4] Dies, H. et al. Electrokinetically-Driven Assembly of Gold Colloids into Nanostructures for Surface-Enhanced Raman Scattering. Nanomaterials (Basel) 10, doi:10.3390/nano10040661 (2020).

[5] Dao, T. C., Luong, T. Q. N., Cao, T. A. & Kieu, N. M. High-sensitive SERS detection of thiram with silver nanodendrites substrate. Adv. Nat. Sci.-Nanosci Nanotechnol 10, 025012, doi:10.1088/2043-6254/ab2245 (2019).

[6] Nowicka, A. B., Czaplicka, M., Kowalska, A. A., Szymborski, T. & Kaminska, A. Flexible PET/ITO/Ag SERS Platform for Label-Free Detection of Pesticides. Biosensors (Basel) 9, 111, doi:10.3390/bios9030111 (2019).

[7] Pham, X. H. et al. 4-Mercaptobenzoic Acid Labeled Gold-Silver-Alloy-Embedded Silica Nanoparticles as an Internal Standard Containing Nanostructures for Sensitive Quantitative Thiram Detection. Int. J. Mol. Sci. 20, 4841, doi:10.3390/ijms20194841 (2019).

[8] Pu, H., Huang, Z., Xu, F. & Sun, D. W. Two-dimensional self-assembled Au-Ag core-shell nanorods nanoarray for sensitive detection of thiram in apple using surface-enhanced Raman spectroscopy. Food Chem. 343, 128548, doi:10.1016/j.foodchem.2020.128548 (2021).

[9] Hussain, A., Sun, D. W. & Pu, H. Bimetallic core shelled nanoparticles (Au@AgNPs) for rapid detection of thiram and dicyandiamide contaminants in liquid milk using SERS. Food Chem. 317, 126429, doi:10.1016/j.foodchem.2020.126429 (2020).
